# A multimodal mentorship intervention to improve surgical quality in Tanzania’s Lake Zone: a convergent, mixed methods assessment

**DOI:** 10.1186/s12960-021-00652-6

**Published:** 2021-09-23

**Authors:** Shehnaz Alidina, Leopold Tibyehabwa, Sakshie Sanjay Alreja, David Barash, Danta Bien-Aime, Monica Cainer, Kevin Charles, Edwin Ernest, Joachim Eyembe, Laura Fitzgerald, Geofrey C. Giiti, Augustino Hellar, Yahaya Hussein, Furaha Kahindo, Benard Kenemo, Albert Kihunrwa, Steve Kisakye, Innocent Kissima, John G. Meara, Cheri Reynolds, Steven J. Staffa, Meaghan Sydlowski, John Varallo, Noor Zanial, Ntuli A. Kapologwe, Caroline Damian Mayengo

**Affiliations:** 1 Program in Global Surgery and Social Change, Harvard Medical School, 641 Huntington Avenue, Boston, MA 02115 USA; 2 Safe Surgery 2020 Project, Jhpiego, Dar es Salaam, Tanzania and Baltimore, MD USA; 3 GE Foundation, Boston, MA USA; 4 Assist International, Dar es Salaam, Tanzania and Ripon, CA USA; 5 Musoma Regional Hospital, Musoma, Tanzania; 6 Department of Surgery and Department of Obstetrics and Gynaecology, Bugando Medical Center, Mwanza, Tanzania; 7 Department of Health, Social Welfare, and Nutrition Service, President’s Office – Regional Administration and Local Government, Dodoma, Tanzania; 8 Nyakahanga Hospital, Karagwe, Tanzania; 9 D-Implement, Dalberg Advisors, Dar es Salaam, Tanzania; 10 Department of Plastic and Oral Surgery, Boston Children’s Hospital, Boston, MA USA; 11 Department of Anesthesiology, Critical Care and Pain Medicine, Boston Children’s Hospital, Boston, MA USA; 12 Department of Curative Services, Ministry of Health, Community Development, Gender, Elderly, and Children, Dodoma, Tanzania; 13 Department of Paediatrics, University of Melbourne, Melbourne, Australia

**Keywords:** Multimodal mentorship intervention, Surgical provider capacity, Surgical quality, Tanzania, Safe Surgery 2020, Workforce

## Abstract

**Background:**

Safe, high-quality surgical care in many African countries is a critical need. Challenges include availability of surgical providers, improving quality of care, and building workforce capacity. Despite growing evidence that mentoring is effective in African healthcare settings, less is known about its role in surgery. We examined a multimodal approach to mentorship as part of a safe surgery intervention (Safe Surgery 2020) to improve surgical quality. Our goal was to distill lessons for policy makers, intervention designers, and practitioners on key elements of a successful surgical mentorship program.

**Methods:**

We used a convergent, mixed-methods design to examine the experiences of mentees, mentors, and facility leaders with mentorship at 10 health facilities in Tanzania’s Lake Zone. A multidisciplinary team of mentors worked with surgical providers over 17 months using in-person mentorship, telementoring, and WhatsApp. We conducted surveys, in-depth interviews, and focus groups to capture data in four categories: (1) satisfaction with mentorship; (2) perceived impact; (3) elements of a successful mentoring program; and (4) challenges to implementing mentorship. We analyzed quantitative data using frequency analysis and qualitative data using the constant comparison method. Recurrent and unifying concepts were identified through merging the qualitative and quantitative data.

**Results:**

Overall, 96% of mentees experienced the intervention as positive, 88% were satisfied, and 100% supported continuing the intervention in the future. Mentees, mentors, and facility leaders perceived improvements in surgical practice, the surgical ecosystem, and in reducing postsurgical infections. Several themes related to the intervention’s success emerged: (1) the intervention’s design, including its multimodality, side-by-side mentorship, and standardization of practices; (2) the mentee–mentor relationship, including a friendly, safe, non-hierarchical, team relationship, as well as mentors’ understanding of the local context; and (3) mentorship characteristics, including non-judgmental feedback, experience, and accessibility. Challenges included resistance to change, shortage of providers, mentorship dose, and logistics.

**Conclusions:**

Our study suggests a multimodal mentorship approach is promising in building the capacity of surgical providers. By distilling the experiences of the mentees, mentors, and facility leaders, our lessons provide a foundation for future efforts to establish effective surgical mentorship programs that build provider capacity and ultimately improve surgical quality.

**Supplementary Information:**

The online version contains supplementary material available at 10.1186/s12960-021-00652-6.

## Background

Recently, the quality and safety of surgical services in African countries has received increasing attention [1–3]. Patients are twice as likely to die after surgery compared to the global average, and postsurgical infection rates are 2 to 10 times higher [2, 4, 5]. Poor surgical quality stems from the convergence of weak health systems, insufficient resources, and workforce challenges. The number of specialists such as surgeons, anesthesiologists, and obstetricians are estimated to be 20–50 times lower in African countries [6] than the recommended minimum needed to provide safe surgical care. (1) While necessary, bolstering the surgical workforce is neither feasible in the short term, nor sufficient on its own [7]. Research in African settings suggests that building the capacity of existing surgical care providers can improve surgical quality [8–11].

The most common method to build healthcare providers’ capacity in low-resource settings is training and supervision [12, 13]. However, research suggests that these alone are not sustainable [14] and do not effectively improve performance or quality [12, 15]. A multipronged strategy incorporating clinical training and practice at the worksite may be more effective [16]. In particular, mentorship, “the process through which an experienced and empathetic person who is proficient in her/his content area teaches and coaches another individual or group of individuals in person and/or virtually to ensure competent workplace performance and provide ongoing professional development” offers a promising strategy for improving surgical provider capacity [17]. Key elements include mutual trust and respect, shared learning objectives, building skills and confidence, and empowerment [18]. Evidence for mentorship’s effectiveness in African healthcare settings [19] includes improved infectious disease management [20–22], integrated management of childhood illnesses [23, 24], and improved quality of laboratory services [25, 26].

Using mentorship to build surgical care provider capacity in low-resource settings is quite recent but [9, 27, 28] holds promise for three reasons. First, mentorship may be more effective at transferring tacit knowledge and skills following initial training [29] through observation, coaching, and practical solutions in the local context. Second, mentorship may result in more sustainable changes, since it allows surgical providers to continue learning in their environment [30], balance learning with routine work [28], and participate in whole-team learning. Finally, mentorship that provides ongoing learning in smaller amounts spread over time (a “low-dose high frequency approach”), has been effective in adult learning [30, 31]; studies have shown that surgical providers in low-resource settings prefer this method [32].

In 2018, a multimodal mentorship component of a Safe Surgery 2020 (SS2020) intervention was implemented to build surgical care provider capacity in 10 health facilities in Tanzania’s Lake Zone. SS2020 had two goals: (1) improve surgical quality processes including safety practices, teamwork and communication, and data quality, and (2) reduce postsurgical infections [8, 9, 27, 33–35]. Mentorship was the final phase of SS2020 and was designed to support technical and non-technical skill development for non-specialist providers to improve surgical quality, following training [8]. In this paper, we report findings of a mixed-methods assessment of the intervention. By evaluating experiences of mentees, mentors, and facility leaders, our study aims to provide policymakers, intervention designers, and practitioners with information on key elements of a successful surgical mentorship program.

## Methods

### Study design

We used a convergent mixed-methods study design [36–38] as recommended by experts for the study of complex interventions [39]. We collected quantitative data from surveys and qualitative data from interviews and focus groups. Together, our analyses provided a fuller understanding of the lived experiences of mentees, mentors, and facility leaders with the mentorship intervention [40, 41]. We followed the consolidated reporting guidelines for qualitative research [42].

### Setting

Our setting was ten SS2020 intervention facilities in the Mara and Kagera regions in Tanzania’s Lake Zone, a focus of national and international efforts, because they are among the most rural and poorest regions [43]. The intervention facilities (health centers, district hospitals, and regional referral hospitals) were selected based on a feasibility assessment conducted by SS2020 partners in 2018 [27]. Non-specialist surgical providers are the backbone of the surgical system, but they work in challenging circumstances: low-provider density and high patient ratios, inadequate surgical infrastructure, and limited training opportunities [44].

### Participants

Our study participants included mentees, mentors, and facility leaders. Mentees were members of the surgical team, including surgical providers, anesthesia providers, theatre nurses, and nurses in postsurgical and postnatal wards. Mentors were a four-person team, including a surgeon or obstetrician/gynecologist, an anesthesia provider, a theatre nurse, and a labor or postoperative ward nurse. Facility leaders included either medical officers-in-charge or hospital matrons or patrons.

### Selection and preparation

In the first year, all four mentors came from Bugando Medical Center (BMC), the zonal referral and teaching hospital in the Lake Zone. One year after implementation, for cost and sustainability, two mentors came from BMC and two from regional facilities. Mentors were selected based on their specialty, clinical experience, and interpersonal skills. BMC mentors participated in leadership and clinical trainings with mentees and received 3 days of training on mentorship. Regional mentors received 3 days of training on mentorship and worked side-by-side with BMC mentors for 6 months.

### Multimodal delivery

A multimodal approach to mentorship (Table 1) was implemented starting June 2018. For in-person mentorship, the mentor team visited each facility bi-monthly for 2 days. Mentors debriefed with surgical teams on their progress with their surgical quality improvement plan—developed after leadership training—and any perioperative and organizational challenges. Next, each mentor focused on one of three tracks: (1) the *clinical track* included side-by-side coaching in the operating room (OR) on surgical skills, safety practices, and teamwork and communication. Mentors also participated in ward rounds and provided hands-on coaching on postoperative care; (2) the *data track* included reviewing patient files for completeness of documentation and quality of care and coaching on monitoring and use of data to improve surgical quality; and (3) the *patient pathway track* examined care from the facility gate to discharge. Mentors debriefed with the surgical team, facility leaders, and Council Health Management Teams, problem-solved, and developed an action plan identifying opportunities and strategies for improvement.

**Table 1:** Safe surgery 2020 multi-modal mentorship intervention

	In-person mentorship	Tele-mentoring (Project ECHO)	Smartphone App (WhatsApp)
Description	On-site mentorship by multidisciplinary mentorship team to surgical team at each facility focusing on technical and non-technical skills	Telementoring sessions using a hub (mentors and international faculty) and spokes (surgical teams at 10 facilities in two regions). Open to all providers at facility	Regional WhatsApp groups including mentors and surgical teams at 5 facilities in the region
Frequency	2 days every 2 months	80-min weekly sessions	As needed over 24 h, 7 days a week
Focus	Mentorship on three tracks: clinical, data and patient pathway Facilitation on development and implementation of the Quality Improvement Action Plan Problem solving on perioperative and organizational challenges Technical support on Facility Accelerator Fund priorities Advocate on surgical priorities with facility and regional leaders	Didactic presentation on safe surgery topics Presentation of a best practice or challenging case by one facility Discussion and feedback on case by peers and mentors Promotion and dissemination of clinical updates and recommendations	Communication by mentors, mentees, and safe surgery program team Advice on management of patients Sharing of monthly postsurgical infection rates and other program indicators by each facility Sharing of successes and challenges
Examples of mentorship activities	Side-by-side mentoring on the Joel Cohen cesarean section technique to reduce postoperative complications Coaching on the SSC in the OR by explaining why something is important and how to perform it effectively Coaching on screening, classification and management of surgical site infections during ward rounds	Presentation and demonstration on standards for sterile processing of surgical instruments Training sessions for hospital technicians and biomedical engineers covering various issues related to hospital equipment maintenance and repair Presentation on how to manage four types of post-partum hemorrhage by mentors, followed by a case presentation by a surgical team and discussion by surgical providers and mentors	Mentee in a remote facility sends a message on a patient’s condition including photos and X-ray or lab results. Mentor provides guidance on patient management Mentors share short video clip and photos demonstrating the B-Lynch procedure for the management of post-partum hemorrhage Mentees submit monthly postsurgical infections rates. Mentors provide advice on strategies for improvement. Peers cheer or send encouraging messages

Telementoring was provided through the Project ECHO (Extension for Community Healthcare Outcomes) platform using a hub and spoke approach; mentors (the hub) partnered with surgical teams (the spokes) to facilitate sharing of knowledge and expertise through videoconferencing [45–49]. Biweekly 80-min telementoring sessions were open to all relevant staff. Mentors shared knowledge on a variety of topics through didactic presentations. Next, surgical teams presented cases on best practices or a challenging case related to that topic, followed by peer-to-peer and mentor feedback. Regional WhatsApp groups were established to facilitate further communication, sharing of knowledge and monthly surgical data, and real-time problem solving.

### Data collection and analysis

#### Data collection

Members of the research team, (SA, MS) with experience in global health and implementation science developed a survey based on the goals of the intervention and from literature on mentorship in African healthcare settings [19]. The 57 items focused on six topics: (1) satisfaction with mentorship; (2) perceived impact; (3) experience with mentorship; (4) important mentor characteristics; (5) challenges to implementing mentorship; and (6) respondent characteristics (Additional file 1). The survey was tested in five health facilities in a neighboring region with surgical providers. A paper-based survey was administered 14 months after the start of the intervention to mentees who were interviewed. No survey identifiers were collected, and no incentives were offered for completion.

Two research assistants (NZ, MS) experienced in qualitative research with master’s degrees in global health conducted in-depth, semi-structured interviews with mentees, mentors, and facility leaders 14 months after initiation of the intervention. To recruit mentees and facility leaders, we identified the necessary stakeholders (one facility leader and two to three members of the surgical team); the facility or surgical team leaders identified the interviewees. Interviews were 30 min and conducted in a private space by a research team member; daily field notes were recorded after. Three protocols were used for mentees, mentors, and facility leaders and included questions about perceived impact, the multimodal approach, which areas of mentorship were most valued, mentee–mentor relationships, and challenges (Additional file 2). Interviews were conducted in English with translation to Kiswahili when required by Tanzanian SS2020 staff, audiotaped and transcribed. No interviewee ended the interview early or declined to participate. Transcripts were checked for accuracy and uploaded to NVivo V.11 (QSR International, Melbourne, Australia) for coding.

A research assistant (MS) conducted one focus group per facility 10 months after telementoring, with daily field notes. Focus groups included a range from 4 to 15 interviewees per site (facility leaders and members of the surgical team) and lasted 30 min. Questions covered perceived impact, most useful areas of mentoring support, what was best delivered through a virtual platform, challenges, and sustainability (Additional file 2).

#### Data analysis

We used descriptive statistics to summarize the percentage of responses on the Likert scale for each survey question. Denominators included the actual number of responses to the question. Statistical testing was not conducted as this was an exploratory study and the sample size was small (*n* = 25). All analyses were performed using Stata version 16.0, (StataCorp LLC. College Station, TX).

Qualitative data were analyzed by three researchers (NZ, DBA, SA) using the constant comparative method to understand the elements of a successful mentorship program [41, 50]. Researchers reviewed three transcripts to arrive at a preliminary codebook and tested it against nine new transcripts. As new data were coded, we compared the new text segments to those previously assigned the same code; codes were refined to ensure validity until no new codes emerged (i.e., theoretical saturation) [51, 52]. Development of the coding structure, coding definitions, and principles used in applying the codes were documented. Inter-rater reliability between two coders was high (*κ* = 0.87) [53], and all 45 transcripts were analyzed using the final codebook. Recurrent and unifying concepts were identified, and the qualitative and quantitative data were merged to identify the major patterns and themes.

## Results

The ten facilities included regional hospitals, district hospitals, and health centers (Table 2). The typical facility was a 101–300 bed government-operated facility performing an average of 90 major surgeries per month. Our response rate for the survey was 82%. Interviewees (*n* = 45) included mentees (62.2%), mentors (17.8%), and facility leaders (20%).

**Table 2:** Characteristics of intervention facilities and participants, 2019

Facility characteristics (*N* = 10) n (%)	
Level of facility	
Health Centers	2 (20)
District Hospitals	6 (60)
Regional Referral Hospitals	2 (20)
Geography	
Rural	5 (50)
Urban	3 (30)
Suburban	2 (20)
Number of inpatient beds	
0–100	3 (30)
101–300	6 (60)
300 +	1 (10)
Average monthly major surgeries per facility	90
Average number of surgical providers per facility	
Surgeons	0.2
Obstetricians/gynecologists	0
Anesthesiologists	0
Medical Officers performing surgery	4.1
Assistant Medical Officers performing surgery	3.7
Non-physicians proving anaesthesia	2.8
Participant characteristics	
Survey (*N* = 25) *n* (%)	
Role	
Surgical provider	11 (39.1%)
Anaesthesia providers	5 (21.7%)
Nurse	7 (30.4%)
Other (facility leader also a surgical provider)	2 (8.7%)
Years in role	
< 1 year	1 (4.3%)
1–3 years	7 (30.4%)
3 + years	14 (60.9%)
Missing	1 (4.3%)
Present for mentorship visits	
< 3	3 (13%)
3 +	18 (78.3%)
Missing	2 (8.7%)
Interviews (N = 45) n (%)	
Role	
Mentees	
Surgical provider	12 (26.7%)
Anaesthesia provider	5 (11.1%)
Nurses	11(24.4%)
Facility leader	9 (20%)
Mentor	8 (17.8%)
Focus Groups (*N* = 10)	
Attendees per focus group (surgical providers, anesthesia providers, facility leaders)	4–15

Below we present quantitative and qualitative results for our study, including perceptions related to the successful impact of mentoring, three themes, and eleven constituent subthemes that contributed to the successful impact and four challenges to implementing mentorship. Table 3 provides illustrative quotations for themes that emerged from the interviews. Quotations have been edited for conciseness.

**Table 3:** Illustrative quotations of themes about features of and challenges to a successful mentorship intervention

Themes and sub-themes	Illustrative Quotations*
Valuable elements of the mentorship intervention	
Multimodality of the mentorship intervention	*They are all valuable, because they all depend on each other and none can stand on behalf of the other. (Surgical Provider, Region 1, Facility 2)*
Supportive side-by-side clinical coaching	*They will perform the first case and we observe and identify the gaps that they have. The second case we all scrub in together -both the mentors and the mentees – and we can perform together so in that way we can impact the knowledge through doing procedures together. (Mentor 1)*
Standardization of practices	*Nowadays we hardly forget the use of the checklist. The theater staff now has a system of preparing the trays for vaginal cleansing prior whereas in the past days that was not present. Therefore, when there is a ruptured membrane you could just order for a tray that has been prepared and sterilized so it has changed the way people work. (Region 1, Facility 3, Facility Leader)*
Useful features of the mentor–mentee relationship	
Relationship-building	*If you have a mentorship relationship it has to be close. Even if you have something it can be easily shared with them. This makes it easy even to share knowledge and interest between mentors and mentees and it can increase the relationship, because you know each other even outside work and if you have something to ask you may communicate with him or her. (Region 2, Facility 4, Surgical Provider)*
Friendliness	*We get feedback from mentees and most of the time, they give us feedback that they benefitted from what we offered them and the way we offered is friendly…and we participated as part of their team. And all this is because we had training before we performed mentorship compared to the formal medical training in Tanzania where there is a gap between a lecturer and a student. (Mentor 5)*
Psychological safety	*It is a fine one, because someone who is not a dictator to you, you may have a conversation and you are able to exchange views. We are comfortable to admit mistake and ask questions and help. (Region 2, Facility 4, Surgical Provider)*
Mentors as part of the surgical team	*The relationship is good, because when they come here we work together and they become like team members of the facility. We work together like team members for quality improvement of services. (Nurse, Region 1, Facility 4)*
Understanding of context	*Mentors were trying to understand the local context and condition of the facility, and we started from the entrance gate. (Region 2, Facility 3, Nurse)*
Helpful characteristics of the mentor	
Non-judgmental feedback	*The mentors were not judgmental as in once you fail they will not judge you. So it is a conversation. They tell you something and you will ask questions and they will correct like ‘do this and you were not supposed to do this’ in a guiding manner and they give a chance to ask questions. (Surgical Provider, Region 2, Facility 4)*
Mentor experience level	*I think when you bring mentors at the facility, they should be senior mentors. For example, I am a senior nurse anaesthetist. When you bring a mentor that is junior to me like someone who has been practicing for less than 6 months in the field then usually cannot add value to me. (Anaesthetist, Region 2, Facility 1)* *The mentors are skilled, the whole team is skilled from the surgeon, anesthetist and the nurse are all skilled so when they come and they face the challenges of the facility they can assist and tackle together with discussion. (Region 1, Facility 1, Surgical Provider)*
Accessibility of the mentor	*They leave behind their numbers and they let people know that they are available so when someone is in trouble they can contact them…There was a time they [surgical team] had a fistula patient, they communicated, and they found a way forward so it has been a team. (Facility Leader, Region 2, Facility 2)*
Challenges to mentorship	
Resistance/lack of buy-in	*The challenge is some of the providers were taking this program like it belonged to those that only attended the [SS2020] training. They were not ready to involve directly on the mentorship program so sometimes you may find that when mentors come to the facility there is high effort used to get all members that are needed for the program. There is still some resistance at the facility in relation to the mentorship program. (Surgical Provider, Region 1, Facility 2)*
Shortage of surgical providers	*Some of the barriers are time, because you may find people have other activities to attend to during the time of presentations/sessions…Another thing is the shortage of staff. Providers may be alone in the ward and it is difficult for them to leave patients and attend the sessions. (Focus Group Discussion, Region 1, Facility 2)*
Mentorship dose	*I think the frequency can increase – at least that they should come monthly and they could stay at least if possible for a week for the mentorship. (Region 2, Facility 1, Surgical Provider)*
Logistical challenges	*The main problem in this is the language barrier, because our country is based very much on Swahili…It would be better to have handouts and translation if possible in Swahili. Sometimes the internet is a problem and it is not stable. (Focus Group Discussion, Facility 1, Region 1)*

*Quotations have been edited for conciseness

### Perceived mentorship impact

Overall, 96% of mentees experienced the intervention as positive, 88% were satisfied, and 100% supported continuing the intervention in the future (Additional file 3). All mentees reported they had changed their surgical practices due to mentorship, and 86% of mentees said their facilities made positive changes (Additional file 4). One leader suggested that, in time, they could become mentor hospitals to other facilities.

**Fig. 1:**
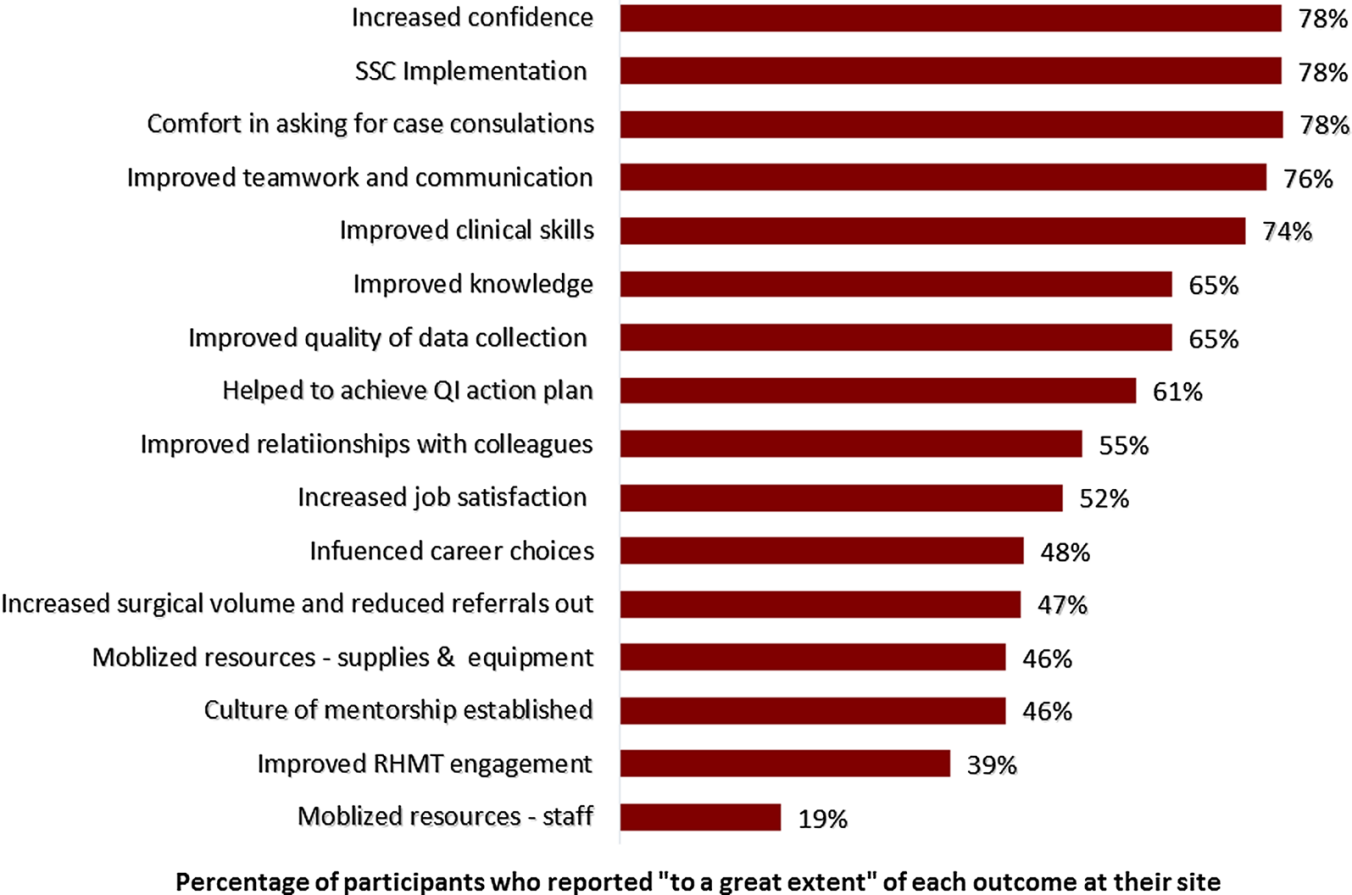
Perceived mentorship impact

Three quarters of all mentees reported mentorship had made a difference “to a great extent” in five areas: confidence, Safe Surgical Checklist (SSC) use, asking for case consultations, teamwork and communication, and clinical skills (Fig. 1). The qualitative data supported these positive survey findings. All participants perceived that mentorship helped mentees improve their confidence, surgical skills, safety practices, teamwork and communication, and safety culture.

Mentors also reported their own clinical skills, surgical practices, and teamwork and communication skills improved from participating in the SS2020 trainings and mentoring. In addition, they reported their leadership skills and confidence had increased.

All participants perceived improvements in the surgical ecosystem, such as better preoperative and postoperative care. In interviews, leaders and surgical providers mentioned increased revenue because of reduced referrals out, increased surgical volume, and improved best practices. Mentees reported improvements had a positive impact on patients, including reduced postsurgical infections, increased safety, improved communication, faster recovery, and reduced decision-to-incision times.

Below we present the themes and subthemes that contributed to the positive impact.

### Valuable elements of the mentorship intervention

#### Multimodality of the mentorship intervention

In interviews, participants reported that the all three platforms—in-person mentorship, telementoring, and WhatsApp (Table 1)—were valuable, because they complemented and reinforced each other, contributing to continuous and deeper learning.

Participants explained that in-person mentorship was optimal for learning tacit knowledge and skills such as how to perform certain surgical techniques. In-person mentorship included side-by-side coaching allowing mentors to role-model important technical and non-technical skills to mentees; a few participants expressed this way of learning was particularly suited to their culture and allowed mentoring to be tailored to the facility’s individual context. Mentors said it allowed them to assess the team environment or the quality of their OR registers, follow-up on progress, problem-solve organizational or perioperative issues, and advocate for surgical priorities with facility, district, or regional management. Mentees reported that they valued the real-time communication with mentors to share experiences. There were two challenges of in-person mentorship: (1) it was resource intensive, and (2) a heavy caseload or the lack of a specific type of case on the day of mentoring may make it difficult to engage in mentorship activities.

Participants noted that telementoring was optimal for learning explicit knowledge, and it expanded their knowledge on safe surgery. It was perceived as time-efficient and less costly, since many people could learn together and mentors did not have to travel. It was also a valuable platform for learning about uncommon cases and convenient to bring in different experts. It was open to everyone which helped to create a learning community on safe surgery and promoted shared learning between surgical teams at different facilities with common contexts and challenges. Logistical challenges included after-work timing of sessions, poor internet connectivity, and lack of translation for English sessions by international faculty. Staff workload prevented some mentees from attending.

WhatsApp was a valuable platform for timely advice on patient management and coordination issues regardless of location. Participants highlighted its convenience, since texts or videos could be reviewed at any time. It also created a sense of community between the surgical teams at various facilities. However, some mentioned issues with confidentiality of information and inaccessibility to WhatsApp as disadvantages.

#### Supportive side-by-side clinical coaching

Mentees prioritized clinical skills highest among all areas of mentorship received. The top three areas mentees prioritized as “greatly important” were also the areas, where they received mentorship “to a great extent”: clinical skills (95.7% said it was “greatly important” and 76.2% said they received it “to a great extent”), SSC implementation (87% and 66.7%, respectively), and utilization of data (78.3% and 59%, respectively) (Additional file 5).

In interviews, mentees prioritized side-by-side clinical mentorship, because it provided practical, efficient, and direct teaching and feedback with tangible improvements, but there were some differences by discipline. Surgical providers, nurses, and leaders valued mentorship on clinical procedures the most, followed by the SSC, while anesthetists valued the SSC the most, followed by the patient pathway.

#### Standardization of surgical practices

Mentors noted that previously, surgical providers and mentors had their own surgical practices at their facilities; after the SS2020 trainings and mentorship, evidence-based practices became standardized. They perceived a culture of following standards improved the quality of care for patients and reduced postsurgical infections.

### Helpful features of the mentee–mentor relationships

#### Relationship building

Mentees and mentors discussed how the mentors first invested in building relationships. Mentees felt that this approach opened the door for mentors to share their knowledge and skills, making it easier for mentees to ask questions and be receptive to feedback for improvement.

#### Friendliness

The term “friendly” was most often used by mentees to describe their relationship with mentors due to their non-hierarchical, collegial approach. Furthermore, they felt that the approach to feedback, where mentors started with positive feedback first before discussing areas for improvement reduced resistance. One mentor attributed their ability to build a friendly relationship with mentees to the leadership training they attended together.

#### Psychological safety

Mentees described their relationship with their mentors as psychologically safe, where they felt comfortable asking questions, seeking help, and admitting mistakes. Mentors said they were also comfortable discussing feedback with mentees. Mentees and mentors said they shared feedback with each other at the end of each visit, through annual mentor evaluations, and at an annual mentorship debriefing session.

#### Mentors as part of the surgical team

Importantly, mentees and facility leaders described their mentors as part of a team working together in the pursuit of surgical quality improvement. One facility leader explained that by becoming part of the team, mentors overcame the common challenge of getting people engaged and it was the sense of being one team that made mentoring a success.

#### Understanding of local context

Mentees and leaders felt that mentors understood their context, such as resources, staffing, and culture, and were able to help with their challenges. They noted that the patient pathway track, where mentors and mentees reviewed services from the gate to discharge provided mentors with a deeper understanding of their facility. This understanding of context allowed mentors to relate to mentees; as a result, knowledge and skills were transferred more easily.

### Useful mentor characteristics

The top three mentor characteristics mentees prioritized as “greatly important” and exhibited “to a great extent” were interpersonal skills (90.4% felt these were “greatly important” and 81% felt mentors exhibited them “to a great extent”), role modeling (82.6% and 65.2%, respectively), and courtesy and respect (81% and 81%, respectively) (Additional file 6). In interviews, participants raised the importance of mentors who provided non-judgmental feedback, had sufficient experience, and were accessible.

#### Non-judgmental feedback

Mentees highly valued the mentors’ nonjudgmental feedback. Mentees mentioned mentors fostered a supportive environment by providing feedback, offered guidance and a chance to ask questions with the goal of learning and improvement.

#### Mentor experience level

When describing attributes, many mentees spoke very positively about the experience level of mentors. However, some felt that their mentors lacked sufficient experience in their discipline or in mentorship and were not able to be as helpful as a result.

#### Accessibility of the mentor

Mentees discussed they could reach out to their mentors outside mentorship visits if they had a difficult surgical case, which was very helpful. One leader provided an example of when the surgical team reached out to their mentors for advice on the care of a patient with a fistula and worked together as a team.

### Challenges to mentorship

Four themes emerged as barriers to successful implementation of mentorship. First, while most mentees had a good attitude towards mentorship and welcomed it, participants mentioned there was resistance from some mentees. Reasons mentioned included: First, mentees initially perceiving it as another supervision program, less buy-in from mentees who had not attended the SS2020 trainings, and difficulty changing practice. Second, most facilities faced a shortage of surgical providers, so mentees were not prepared when mentors arrived or needed to attend to routine duties during mentorship; afterhours sessions were also unpaid. Third, while some mentees felt the mentorship ‘dose’ was just right, others felt the frequency and duration of mentorship was insufficient. Finally, there were logistical challenges including communication around timing of the mentorship visits, after work hours of telementoring sessions, poor internet connectivity, and language barriers with international faculty.

## Discussion

We examined a multimodal approach to mentorship as part of a safe surgery intervention to improve surgical quality in Tanzania’s Lake Zone to distill lessons on its key elements of success (Box 4).

**Box 4:** Lessons for implementing a successful mentorship intervention to strengthen surgical services in low-resource settings

Intervention design	
• A multimodal mentorship intervention design using both in-person and virtual platforms can support different types of learning (e.g., tacit or explicit). The different platforms complement and reinforce each other, contributing to continuous and deeper learning	
• Mentorship is optimized when it is part of a *multicomponent intervention*. Training mentees and mentors on evidence-based practices before mentorship ensures that everyone is working to implement the same standards for safe surgery	
• A *team-based* approach to mentorship can provide discipline-specific mentorship (e.g., nurses mentoring other nurses), and reinforce a culture of shared learning	
• To improve the intervention, there should be opportunities for *reflection and learning*. Incorporating time for bi-directional feedback, such as debriefing after each visit, at annual meetings, and evaluations can strengthen future intervention design	
Mentors	
• *Selecting the right mentors* is key. Subject matter expertise and strong interpersonal and communication skills are crucial. Selecting local mentors can facilitate cultural congruence and an understanding of context, relatability, and language. Local mentors can also train new surgical providers more frequently and engage in peer-to-peer learning to diffuse knowledge quickly and continuously	
• *Preparation of mentors* should cover subject matter expertise, change management skills, and mentorship skills, such as relationship-building, communication and feedback, and effective teaching. Pairing junior mentors with experienced mentors can also be considered to strengthen mentorship skills and confidence	
• *Mentorship requires resources*. Mentors need protected time away from clinical work to prepare and conduct mentorship visits as well as resources for coordination, training, and support. Options for incentivizing mentors through compensation, continuing education credits or other incentives like certification should be considered	
Implementation	
• A *Quality Improvement Action Plan* can facilitate a shared understanding about the overall improvement goals of the intervention. An action plan can lay out a clear strategy (e.g., specific actions, responsibilities, timing and means of verification) and can provide a framework for assessing progress and setting goals for the next visit	
• *Buy-in* from the surgical team is essential before starting the mentorship intervention; they must understand the goals. It is especially important to address those who are less ready to change. Whole-site orientation and training and engaging facility leaders in mentorship can increase buy-in	
• *Leadership support and engagement* from facilities, district and regional leaders is necessary for success. Leaders can support staff in implementing mentoring activities, release staff time, and assist in setting up QI systems. Furthermore, leadership support is crucial in sustaining surgical quality improvement. Mentorship cannot work if leadership is not receptive to it	
• *Time constraints* must be considered for mentees and mentors. Health facilities in low-resource settings are often faced with staff shortages. Mentors also have competing work and personal demands. Therefore, implementation must consider providing surgical providers the time to learn and improve, timing of sessions, lowering work burden, and revamping tools for efficiency for both mentees and mentors	
Sustainability	
• Building a *culture of mentorship* is necessary for sustainability. Mentorship is a promising approach for scaling surgical quality and requires policy support to institutionalize it. Mentorship should be incorporated in the safe surgery space, linked to continuing professional development systems, and should be incorporated in the District plans and budget. Training a pool of local multidisciplinary mentors is critical for cost effectiveness and sustainability	

Our results suggest that mentorship can act as a vehicle to build capacity of surgical providers in low-resource settings. Mentees, mentors, and facility leaders perceived improvements in surgical practice and teamwork in the OR, strengthening of the surgical ecosystem, and a reduction in postsurgical infections; this is supported by our quantitative findings [8]. We attribute the success of the intervention to its multimodal design, collaborative relationships between mentees and mentors, and its grounding in local context.

Most studies examining mentorship in surgery are based in high-income countries [29]. While research on surgery in low-resource settings is sparse [19, 28, 54], our findings are consistent with the emerging evidence about elements of successful mentorship. Mentorship that follows training, uses a side-by-side approach, has high-quality mentee–mentor relationships, understands the local context, and provides nonjudgmental feedback leads to success in low-resource settings [17–19, 24, 55].

A key feature of the intervention was its multimodality. Our goal was to improve surgical quality, which required the development of technical and non-technical skills, behavior changes, and new ways of thinking. A multimodal mentorship approach offered a flexible journey to transform surgical practice and culture. In-person mentorship was optimal for transferring tacit knowledge and skills, telementoring for explicit knowledge, and WhatsApp for real-time problem-solving. This approach allowed for a balance of work and learning, attention to contextual factors, and integration of knowledge and practice. Our findings contribute to the evidence on the effectiveness of multimodal mentorship approaches [56–60].

Relationship building between mentees and mentors was also important for success. To build relationships, mentors presented themselves to mentees as a helper, not as an instructor or a manager. They created a safe, nonjudgmental environment, where mentees felt comfortable sharing their challenges and were receptive to feedback. Mentors from regional and local hospitals understood the local context and were perceived as colleagues. A team-based approach which provides discipline-specific mentorship (e.g., nurses mentoring other nurses), can also facilitate technical congruence [18]. Properly training mentors was crucial; during the COVID-19 pandemic mentorship became fully virtual, which required a different skill set than in-person mentoring. Preparation covered how to structure sessions, develop activities, and effectively facilitate virtual sessions.

Literature points to the importance of bridging the knowing-doing gap [34, 61–63]. While most mentees had a positive attitude towards mentoring, there was some resistance from others. It is important to cultivate buy-in by orienting the surgical team to the goals of mentorship, inviting their input on priorities, and training the whole team. Leadership engagement is also critical for successful and sustainable mentorship. Leaders can signal its priority, release staff time for mentorship activities, and create structures that build learning capacity [33, 64–67].

Sustainability requires policy support and a culture of mentorship. It is necessary to engage with national health system leaders to develop policy that integrates surgical mentorship into the existing health system and incorporates it in district plans and budget, including training and compensation for mentors. The concept of mentorship should be introduced during surgical training, incorporated into mentors’ regular responsibilities, and linked to continuing professional development [17].

Future research should consider experimental and longitudinal designs to identify which features of the mentorship program are associated with significant improvements in surgical quality. In addition, studies should examine the cost-effectiveness of multimodal mentorship approaches in comparison to unimodal approaches. Research should also consider what contextual factors are important for successful surgical mentorship in African settings.

Our study has limitations. Our findings should be confirmed in diverse contexts to generalize results. Our survey did not undergo formal testing to establish reliability and validity. Data were self-reported, so the findings may appear more positive due to social desirability bias [68], and language barriers may have limited the discussion. The qualitative data from interviews may be confounded by participant experiences with other SS2020 intervention components. A key strength of our research was its mixed methods approach, which allowed for a deeper understanding of participants’ experiences. Furthermore, the implementation facilitator and barriers found here offer a valuable learning opportunity as they likely apply to mentorship interventions in low-resource settings more generally.

## Conclusions

In this paper, we report on the findings of a mixed-methods assessment of a multimodal mentorship intervention with surgical providers in Tanzania’s Lake Zone to improve surgical quality. We found that a multimodal design, high-quality mentee–mentor relationships, and understanding of local context can optimize mentorship. The themes identified offer insights and lessons that can inform policy makers, intervention designers, and practitioners about successful implementation of surgical mentorship interventions. Future research should examine the cost-effectiveness of multimodal mentorship approaches and the contextual factors that are important to optimize surgical mentorship in African settings.

## Supplementary Information

**Additional file 1:** Mentorship survey.

**Additional file 2:** Interview protocols for mentees, mentors and facility leaders and focus group discussion questions.

**Additional file 3:** Overall experience, overall satisfaction and support for continuation of the mentorship program.

**Additional file 4:** Changes made resulting from the mentorship program.

**Additional file 5:** Areas of Mentorship desired and received by mentees.

**Additional file 6:**Mentor characteristics desired by mentees and exhibited by mentors.

## Data Availability

The data set analyzed during the current study is available from the corresponding author on reasonable request. All requests for data must be approved by the Tanzania Ministry of Health, Community Development, Gender, Elderly and Children, in accordance with the data sharing agreement.

## Abbreviations

SS2020: Safe Surgery 2020
SSC: Surgical Safety Checklist
Project ECHO: Project Extension for Community Healthcare Outcomes
OR: Operating Room
BMC: Bugando Medical Center
