# Supplementary material for: A multimodal mentorship intervention to improve surgical quality in Tanzania’s Lake Zone: a convergent, mixed methods assessment
Source: Hum Resour Health. 2021 Sep 23;19:115. doi: 10.1186/s12960-021-00652-6 (PMC8458007; doi:10.1186/s12960-021-00652-6)
Supplement: Supplementary file 1 — Additional file 1: Mentorship survey. [file 12960_2021_652_MOESM1_ESM.docx]

**Additional File 1 – Mentorship Survey**

**Safe Surgery 2020 - Tanzania Mentorship Survey**

The goal of the Safe Surgery 2020 initiative is to reduce preventable deaths from surgically-treatable conditions by improving safe, timely, and affordable access to surgical care. Safe Surgery 2020 partners include Dalberg, a strategy advisory firm, Jhpiego, an international health NGO, Harvard Medical School Program in Global Surgery and Social Change (PGSSC), a research institution, and Assist International, a humanitarian organization. We are funded by the GE Foundation.

We are conducting a survey of participants involved in the Safe Surgery 2020 initiative to obtain feedback on the mentorship program. Your response will help us to understand your perception of the mentorship program related to areas of mentoring, experience with your mentor, impact made by the mentorship program, and how the program can improve.

Completing this survey should take **less than 30 minutes**.

We will collect your answers **confidentially** and will not share them with anyone else in an identifiable fashion. No one at this facility will have access to your responses; they will only see summary reports.

Participation in this survey is **completely voluntary.** If you choose not to complete this survey, it will have no effect on your employment and no one will know that you declined to participate.

The completion of this survey **implies your consent** to participate.

Participants will **not receive any compensation** for their involvement in completing this survey.

You may **skip any questions** that you do not feel comfortable answering.

**Please do not write your name on this survey**

Survey responses will be analyzed by researchers in the Program in Global Surgery and Social Change at Harvard Medical School.

Thank you very much for taking this survey. Your participation is very important to us. If you have any questions regarding this project or survey, please contact Shehnaz Alidina at shehnaz_alidina@hms.harvard.edu

1. **Respondent and facility characteristics**

*The purpose of this section of the survey is to collect background information on you as a surgical provider, the facility in which you work, and the extent to which you were present for mentor visits.*

1. What is the name of your health facility?

__________________________________________________

1. What is your professional role at this facility?

- Hospital leader: _______________________________________________
- Surgical provider: ______________________________________________
- Anesthesia provider­: ____________________________________________
- Nurse: _______________________________________________________
- Quality improvement officer: _____________________________________
- Other: ________________________________________________________

1. How many years have you been in practice in your role?
   - Less than 1 year
   - 1 to 2 years
   - 2 to 3 years
   - More than 3 years
2. Did you participate in the Safe Surgery 2020 leadership training conducted in May 2018?
   - Yes
   - No
3. Did you participate in the Safe Surgery 2020 clinical training conducted in September 2018?
   - Yes
   - No
4. How many Safe Surgery 2020 mentor visits were you present for?
   - 0 [**please don’t continue with this questionnaire**]
   - 1
   - 2
   - 3
   - More than 3
5. **Areas of mentoring support**

*The purpose of this section of the survey is to understand your perception on the importance of specific areas of mentoring as well as your experience receiving mentoring support on these areas through our mentorship program.*

**From your perspective**, please indicate: 1) the importance of each of the mentoring areas in your opinion and 2) the extent to which your team received mentoring support in each of the areas.

| ***Section B: Areas of mentoring support*** | | | | | | |
| --- | --- | --- | --- | --- | --- | --- |
| **B1. Clinical skills training and knowledge enhancement** | | | | | | |
| Importance of this mentoring area | - Not at all important | - Slightly important | - Moderately important | - Greatly important |  |  |
| Extent to which your team received mentoring in this area | - To no extent | - To a small extent | - To a moderate extent | - To a great extent |  |  |
| **B2. Implementation of the Safe Surgical Checklist** | | | | | |  |
| Importance of this mentoring area | - Not at all important | - Slightly important | - Moderately important | - Greatly important |  |  |
| Extent to which your team received mentoring in this area | - To no extent | - To a small extent | - To a moderate extent | - To a great extent |  |  |
| **B3. Prioritizing surgical problems and framing problems appropriately** | | | | | |  |
| Importance of this mentoring area | - Not at all important | - Slightly important | - Moderately important | - Greatly important |  |  |
| Extent to which your team received mentoring in this area | - To no extent | - To a small extent | - To a moderate extent | - To a great extent |  |  |
| **B4. Review and modification of the Quality Improvement plan** | | | | | |  |
| Importance of this mentoring area | - Not at all important | - Slightly important | - Moderately important | - Greatly important |  |  |
| Extent to which your team received mentoring in this area | - To no extent | - To a small extent | - To a moderate extent | - To a great extent |  |  |
| **B5. Data collection and reporting** | | | | | |  |
| Importance of this mentoring area | - Not at all important | - Slightly important | - Moderately important | - Greatly important |  |  |
| Extent to which your team received mentoring in this area | - To no extent | - To a small extent | - To a moderate extent | - To a great extent |  |  |
| **B6. Utilization of data** | | | | | |  |
| Importance of this mentoring area | - Not at all important | - Slightly important | - Moderately important | - Greatly important |  |  |
| Extent to which your team received mentoring in this area | - To no extent | - To a small extent | - To a moderate extent | - To a great extent |  |  |

| ***Section B: Areas of mentoring support continued*** | | | | | |
| --- | --- | --- | --- | --- | --- |
| **B7. Resource mobilization (mobilizing equipment and supplies)** | | | | | |
| Importance of this mentoring area | - Not at all important | - Slightly important | - Moderately important | - Greatly important |  |
| Extent to which your team received mentoring in this area | - To no extent | - To a small extent | - To a moderate extent | - To a great extent |  |
| **B8. Mobilization of staff** | | | | | |
| Importance of this mentoring area | - Not at all important | - Slightly important | - Moderately important | - Greatly important |  |
| Extent to which your team received mentoring in this area | - To no extent | - To a small extent | - To a moderate extent | - To a great extent |  |

B9. Please write in any additional areas in which you received mentoring

|  |
| --- |
|  |
|  |
|  |
|  |

1. **Perceptions of program**

*The purpose of this section of the survey is to understand your perception and experience with the program.*

C1. Please indicate your experience with the mentorship program overall:

- Very negative
- Somewhat negative
- Neutral
- Somewhat positive
- Very positive
- Not applicable (have not received or been present for mentorship visits)

1. **Perceptions of mentors and mentor-mentee relationships**

*The questions in this section are intended to understand what you value in a mentor and to learn about your experience with your mentor. The purpose of this section is* ***not*** *to critique or evaluate mentors in the program.*

The following table includes various characteristics, qualities, and abilities. **From your perspective**, please indicate: in general how important you believe each of the elements are for an effective mentor, and in your experience with your Safe Surgery 2020 mentor(s), the extent to which your mentor(s) exhibited the characteristics or qualities.

| ***Section D: Perceptions of mentors and mentor-mentee relationships*** | | | | |
| --- | --- | --- | --- | --- |
| **D1. Trust** | | | | |
| Importance of this mentor characteristic | - Not at all important | - Slightly important | - Moderately important | - Greatly important |
| Extent to which your mentor(s) exhibited this characteristic | - To no extent | - To a small extent | - To a moderate extent | - To a great extent |
| **D2. Courtesy and respect** | | | | |
| Importance of this mentor characteristic | - Not at all important | - Slightly important | - Moderately important | - Greatly important |
| Extent to which your mentor(s) exhibited this characteristic | - To no extent | - To a small extent | - To a moderate extent | - To a great extent |
| **D3. Teaching skills** | | | | |
| Importance of this mentor characteristic | - Not at all important | - Slightly important | - Moderately important | - Greatly important |
| Extent to which your mentor(s) exhibited this characteristic | - To no extent | - To a small extent | - To a moderate extent | - To a great extent |
| **D4. Interpersonal skills** | | | | |
| Importance of this mentor characteristic | - Not at all important | - Slightly important | - Moderately important | - Greatly important |
| Extent to which your mentor(s) exhibited this characteristic | - To no extent | - To a small extent | - To a moderate extent | - To a great extent |
| **D5. Constructive feedback** | | | | |
| Importance of this mentor characteristic | - Not at all important | - Slightly important | - Moderately important | - Greatly important |
| Extent to which your mentor(s) exhibited this characteristic | - To no extent | - To a small extent | - To a moderate extent | - To a great extent |

| ***Section D: Perceptions of mentors and mentor-mentee relationships continued*** | | | | |
| --- | --- | --- | --- | --- |
| **D6. Knowledgeable in the field** | | | | |
| Importance of this mentor characteristic | - Not at all important | - Slightly important | - Moderately important | - Greatly important |
| Extent to which your mentor(s) exhibited this characteristic | - To no extent | - To a small extent | - To a moderate extent | - To a great extent |
| **D7. Aware of mentee’s work environment and local context** | | | | |
| Importance of this mentor characteristic | - Not at all important | - Slightly important | - Moderately important | - Greatly important |
| Extent to which your mentor(s) exhibited this characteristic | - To no extent | - To a small extent | - To a moderate extent | - To a great extent |
| **D8. Role model in providing surgical services** | | | | |
| Importance of this mentor characteristic | - Not at all important | - Slightly important | - Moderately important | - Greatly important |
| Extent to which your mentor(s) exhibited this characteristic | - To no extent | - To a small extent | - To a moderate extent | - To a great extent |
| **D9. Belief in capabilities and potential of mentee** | | | | |
| Importance of this mentor characteristic | - Not at all important | - Slightly important | - Moderately important | - Greatly important |
| Extent to which your mentor(s) exhibited this characteristic | - To no extent | - To a small extent | - To a moderate extent | - To a great extent |

1. **Difference made by mentoring**

*The purpose of this section of the survey is to understand the perceived impact of the mentorship program. The questions in this section are related to the level of impact made by each area of mentor support, whether or not changes were made as a result of the program, and if the mentorship program had an impact on your personal life.*

Please indicate **from your perspective** the extent to which the following areas were impacted as a result of the mentorship program:

| ***Section E: Impact of mentoring program*** | | | | |
| --- | --- | --- | --- | --- |
| **E1**. To what extent do you feel comfortable asking for case consultations with your mentor? | - To no extent | - To a small extent | - To a moderate extent | - To a great extent |
| **E2**. To what extent did the knowledge of surgical team increase? | - To no extent | - To a small extent | - To a moderate extent | - To a great extent |
| **E3**. To what extent did clinical skills of surgical team improve? | - To no extent | - To a small extent | - To a moderate extent | - To a great extent |
| **E4**. To what extent did confidence of surgical team in surgical skills increase? | - To no extent | - To a small extent | - To a moderate extent | - To a great extent |
| **E5**. To what extent did the implementation of the Safe Surgical Checklist improve? | - To no extent | - To a small extent | - To a moderate extent | - To a great extent |
| **E6**. To what extent did communication and teamwork improve? | - To no extent | - To a small extent | - To a moderate extent | - To a great extent |
| **E7**. To what extent did team spirit of the surgical team improve? | - To no extent | - To a small extent | - To a moderate extent | - To a great extent |
| **E8**. To what extent did the program help to achieve a quality improvement action plan? | - To no extent | - To a small extent | - To a moderate extent | - To a great extent |
| **E9**. To what extent did quality data collection improve? | - To no extent | - To a small extent | - To a moderate extent | - To a great extent |
| **E10**. To what extent did strategies to increase surgical volume improve? | - To no extent | - To a small extent | - To a moderate extent | - To a great extent |
| **E.11 To what extent did strategies to decrease referrals out improve?** |  |  |  |  |
| **E12**. To what extent did RMHT engagement and support improve? | - To no extent | - To a small extent | - To a moderate extent | - To a great extent |
| **E13**. To what extent did mentors help with community mobilization? | - To no extent | - To a small extent | - To a moderate extent | - To a great extent |
| ***Section E: Impact of mentoring program continued*** | | | | |
| **E13**. To what extent did mentors help with resource mobilization? | - To no extent | - To a small extent | - To a moderate extent | - To a great extent |
| **E14**. To what extent did the program influence hospital management or RHB to deploy additional staff? | - To no extent | - To a small extent | - To a moderate extent | - To a great extent |
| **E15.** To what extent did a culture of mentorship establish? (new traditions, norms because of the mentorship program) | - To no extent | - To a small extent | - To a moderate extent | - To a great extent |

Please indicate whether or not changes you individually or your hospital made as a result of the mentoring program. If changes were made, please briefly describe in the space provided:

| **E14**. Did your surgical team make changes in how it practices surgical care as a result of the mentorship program? | - No | - Yes |
| --- | --- | --- |
|  | *Please explain your answer:* | |
| **E15**. Did your facility make any changes as a result of the mentorship program? | - No | - Yes |
|  | *Please explain your answer:* | |

Please indicate the extent to which the following areas of your personal life were impacted as a result of the program:

| **E16.** To what extent did relationships with your colleagues beyond work improve? | - To no extent | - To a small extent | - To a moderate extent | - To a great extent |
| --- | --- | --- | --- | --- |
| **E17.** To what extent did your job satisfaction increase? | - To no extent | - To a small extent | - To a moderate extent | - To a great extent |
| **E18.** To what extent did the program influence your career choices or interest in career development? | - To no extent | - To a small extent | - To a moderate extent | - To a great extent |

1. **Areas for improvement**

*The purpose of this section of the survey is to allow participants to provide feedback on areas of improvement.*

Please indicate whether or not the following areas of the program needs improvement. If marked “Needs improvement”, please briefly describe how the area could improve in the space provided:

| ***Section F: Areas for improvement*** | | |
| --- | --- | --- |
| **F1.** Frequency of the mentorship visits | - Needs improvement | - Does not need improvement |
|  | *Please briefly describe:* | |
| **F2.** Duration (i.e. time spent) of the mentoring visits | - Needs improvement | - Does not need improvement |
|  | *Please briefly describe:* | |
| **F3.** Structure of the program | - Needs improvement | - Does not need improvement |
|  | *Please briefly describe:* | |

| ***Section F: Areas for improvement continued*** | | |
| --- | --- | --- |
| **F4.** Team composition | - Needs improvement | - Does not need improvement |
|  | *Please briefly describe:* | |
| **F5.** Relationship between mentors and providers | - Needs improvement | - Does not need improvement |
|  | *Please briefly describe:* | |
| **F6.** Areas of mentoring | - Needs improvement | - Does not need improvement |
|  | *Please briefly describe:* | |
| **F7.** Communication between program participants | - Needs improvement | - Does not need improvement |
|  | *Please briefly describe:* | |

In the space provided below, please describe any other areas of improvement of the mentorship program:

|  |
| --- |
|  |
|  |
|  |

1. **Facilitators and barriers**

*The purpose of this section of the survey is to allow participants to provide feedback on facilitators and barriers of the program.*

**G1.** Facilitators of the program are the people or things that may have made it easier to implement the mentoring program or made the program more successful. Please indicate which of the following were the most important facilitators that helped implement the mentoring program and provide any examples you may have (select all that apply):

- Structuring of mentoring program (e.g. comprehensive focus of the mentoring program; availability of a mentoring team, clear goals and expectations, frequency of visits, sufficient contact time, etc.): ______________________________________________________________________________________________________________________________________________________
- Feedback and assistance (e.g. Feedback on quality improvement plan, problem-solving, accountability through the mentorship program, etc.) ______________________________________________________________________________________________________________________________________________________
- Motivating factors (e.g. commitment and dedication of surgical team to services strengthening and to working with a mentoring team): ______________________________________________________________________________________________________________________________________________________
- Enabling environment (e.g. top leadership support for mentorship; time available for mentorship within the department, etc.): ______________________________________________________________________________________________________________________________________________________
- Other: ______________________________________________________________________________________________________________________________________________________

**G2.** Barriers of the program

Barriers of the program are the circumstances or obstacles encountered that may have prevented the mentoring program from being as successful as it could have been. Please indicate which of the following were the most significant barriers to implementing the mentoring program and provide any examples you may have (select all that apply):

- Communication: ______________________________________________________________________________________________________________________________________________________
- Feedback: ______________________________________________________________________________________________________________________________________________________
- Documentation: ______________________________________________________________________________________________________________________________________________________
- Contact time: ______________________________________________________________________________________________________________________________________________________
- Agenda of visits: ______________________________________________________________________________________________________________________________________________________
- Planning of visits: ______________________________________________________________________________________________________________________________________________________
- Attrition of mentors: ______________________________________________________________________________________________________________________________________________________
- Other: ______________________________________________________________________________________________________________________________________________________

1. **Satisfaction**

*The purpose of this section of the survey is to understand your satisfaction with the program and whether or not you support the continuation of the program.*

**H1**. Please indicate the extent to which you are satisfied or dissatisfied with the mentorship program:

- Very dissatisfied
- Somewhat dissatisfied
- Neutral
- Somewhat satisfied
- Very satisfied

**H2**. Please indicate the extent to which you support the continuation of the mentorship program:

- Completely oppose
- Moderately oppose
- Neutral
- Moderately support
- Completely support

**H3.** Please explain your reasoning for opposing or supporting the continuation of the mentorship program:

|  |
| --- |
|  |
|  |
|  |
|  |

1. **Additional feedback**

*The purpose of this section of the survey is to provide any additional feedback that was not captured in the survey or that you would like to elaborate on from an earlier section.*

In the space provided below, please provide any additional comments, feedback, questions, or concerns you may have regarding the mentorship program:

|  |
| --- |
|  |
|  |
|  |
|  |
|  |
|  |
|  |
|  |
|  |
|  |
|  |
|  |
|  |
|  |
|  |
