# Supplementary material for: A multimodal mentorship intervention to improve surgical quality in Tanzania’s Lake Zone: a convergent, mixed methods assessment
Source: Hum Resour Health. 2021 Sep 23;19:115. doi: 10.1186/s12960-021-00652-6 (PMC8458007; doi:10.1186/s12960-021-00652-6)
Supplement: Supplementary file 2 — Additional file 2: Interview protocols for mentees, mentors and facility leaders and focus group discussion questions. [file 12960_2021_652_MOESM2_ESM.docx]

**Additional File 2 - Interview and Focus Group Protocols**

**Interview Protocol – Mentees**

**Background**

1. Can you tell me about your position at the facility? How many years have you worked in this position?
2. Have you had a visit from a mentor in the last 6 months? How many visits have you been present for?
3. **OPTIONAL**: *Has your facility ever undertaken any type of internal mentorship? If so, could you describe your experience.*
   - 1. *For how long did you continue this internal mentorship?*
     2. *Did you evaluate if this internal mentorship benefited your facility? If so, how, and what did you find out?*

**Safe Surgery 2020 Initiative Mentoring Intervention**

1. Why was a mentorship program needed for your facility?
2. In your own words, can you describe the mentorship intervention? (Prompt for process, timeline, structure, SS2020 purpose)

**Areas of mentoring support**

1. In what areas have you received mentoring support? (*if answered by participant, omit from questioning)*
   1. Can you provide an example or description of each area you have received support in?
   2. Of the areas of mentoring discussed, which would you say are the most and least valuable and why?

**OPTIONAL:** *Was mentorship more beneficial for providers in some roles? If so, what skills do you think these providers were able to hone in particular?*

**Perceptions of program, mentors, mentor relationships, and experiences**

Can you describe some characteristics that made your mentor effective? (Prompt for traits and awareness of local context and conditions)

- 1. **PROBE FOR**: Relationship with mentor (relationship dynamics, style of mentoring, comfort and trust), and what worked best for them and their mentor e.g. style of mentorship (if not mentioned before, means of communication, feedback)

1. Can you describe the general attitude around adopting the mentoring program in your facility? (Prompt for acceptance or resistance by some types of providers/all providers, culture change, culture of mentoring)

**Difference made by mentoring**

1. In what ways do you think the mentoring program is contributing to the strengthening of surgical and anesthesia services? (e.g. facility benefits, provider skill benefits, strengthening relationships, etc.) *(if answered by participant, omit from questioning)*

Has mentorship impacted your team dynamics? If so, how? (Probe for freedom to ask questions, open communication etc.)

1. What changes have been made as a result of the mentoring program? (e.g. changes in your professional practice, changes at the facility level)
2. **OPTIONAL:** *Has the program had any impact on your personal life?*
3. In what other ways could mentoring support your facility? (e.g. personally, professionally, work environment-related)

**In-Person Mentoring, Project Echo, and WhatsApp Groups**

1. Have you attended a Project ECHO session?
2. How does Project ECHO compare to the in-person mentorship?
3. Can you please tell me about the WhatsApp Groups you have been using for peer to peer mentoring?
   1. What types of things do you discuss on the WhatsApp Group?
   2. What do you find useful about it?
4. What are the opportunities where the different types of mentoring (in person, Project ECHO, peer-to-peer mentoring (WhatsApp Group)) can complement one another?

**PROBE FOR:**

1. Which mentoring activities are most effective through in-person mentorship?
2. Which types of mentoring activities are most effective through Project Echo? Peer-to-peer mentoring?
3. Do you think all are needed?
4. Do you think the three types of mentorship should be implemented at the same time or one at a time? If it is the latter, in what order should they be implemented?

**Strengths, challenges, areas for improvement, and lessons learned**

1. What would you say are the strengths of the program? (probe for facilitators)

a) Tell me one thing that you especially liked about the mentorship program.

1. What are the areas in which the program could improve? (probe for barriers)

a) If there was one thing you could change about the program what would it be?

**Satisfaction**

How long do you think mentorship should continue for your facility?

1. Can you describe your overall experience with the mentorship program?

**Closing remarks**

1. Is there anything else you would like to add that has not been covered in the interview?

**Interview Protocol – Mentors**

**Background**

1. Can you tell me about your position at this facility? How many years have you worked in this position?
2. Did you participate in Jhpiego’s mentorship and clinical training*?*
3. How many mentoring visits have you participated in?

**Safe Surgery 2020 Initiative Mentoring Intervention**

1. Can you describe your understanding of what mentorship is? How is it different than supervision?
2. In your own words, can you describe the mentorship intervention? (Prompt for goals and priorities, process, timeline, structure, SS2020 purpose)

**PROBE FOR:**

- 1. Can you describe to me what you do as a mentor from the time you enter a facility?
  2. How long are the visits? How often have you had the visits over the last six months? How many surgical staff members do mentors typically work with?

**Areas of mentoring support**

1. Can you describe the areas of mentoring that the program covers in the facility?

**PROBE FOR:**

- 1. Which areas would you say are the most valuable to the surgical teams/ you mentor? Which areas would you say are the least valuable?
  2. Are the mentoring efforts focused inside the facility or also outside the facility (e.g. discussing need for additional resources with RHMT) (*inquire into ecosystem of facility*)

**In person mentoring, Project Echo and WhatsApp Group**

1. How does Project ECHO compare to the in-person mentorship?
2. Can you please tell me about the WhatsApp Groups you have been using for peer-to-peer mentoring?
   1. What types of things do you discuss on the WhatsApp Group?
   2. What do you find useful about it?
3. What are the opportunities where the different types of mentoring (in person, Project ECHO, peer-to-peer mentoring (WhatsApp Group) can complement one another?

Probes:

1. Which mentoring activities are most effective through in-person mentorship?
2. Which types of mentoring activities are most effective through Project Echo? Peer-to-peer mentoring?
3. Do you think all are needed?
4. Do you think the three types of mentorship should be implemented at the same time or one at a time? If it is the latter, in what order should they be implemented?

**Perceptions of program, mentors, mentor relationships, and experiences**

1. Why did you want to be a mentor?
2. How well did the leadership/clinical/mentoring training prepare you for your role as a mentor? Is there anything that should be added or changed to strengthen mentor preparation and support?
3. What characteristics do you think your surgical staff would use to describe you as a mentor?
4. How would you describe your relationships with the surgical staff you mentor?
   1. How comfortable are you in providing mentoring support? Do you feel confident in your skills?
   2. Are surgical staff members willing to seek help from you and admit mistakes?

Do different members of the staff react differently to mentorship? Are nurses/anesthetists more or less attentive than surgeons? Do you have to use different conversation styles, talking points for different types of surgical providers?

1. What is your style of mentoring? *(if answered in previous inquiry, omit question)*

a) Can you describe the ways in which you communicate with the surgical staff?

1. How do you provide feedback?
2. Can you describe the general attitude around adopting the mentoring program in *your facility*?
3. How did the mentors react to their new responsibility? Did you feel the same way?
4. How do you evaluate whether your mentees have learnt/mastered a new skill?

**Difference made by mentoring**

1. In what ways do you think the mentoring program is contributing to strengthening the surgical and anesthetic services at the facilities? (e.g. Facility benefits, provider skill benefits)

**PROBE FOR:**

- 1. Can you give me an example of one change that has been implemented at a facility as a result of your mentorship? [Have you seen changes in staff (e.g. capabilities, confidence, team functioning etc), facility (e.g. quality of care, patient centeredness etc.) or system processes (e.g. data quality)?]

1. What has been the most significant change or impact you have seen in your surgical staff as a result of this program?

Do you think every facility that you visited had different mentoring needs? Do you think different facilities need different mentoring programs? Or does the same program work for everybody?

1. **OPTIONAL:** *What changes are still needed in the facilities you mentored? (e.g. management, work environment-related, improved quality of care)*

**Strengths, challenges, areas for improvement, and lessons learned**

1. From your perspective as a mentor, what would you say are the strengths of the program?
2. Tell me one thing that worked well for you in the mentorship program in its current form).

What would you say are the challenges associated with the program? (e.g. facility challenges, foreseen challenges with sustainability or continuation of the program)?

1. What are the areas in which the program could improve? ( If there was one thing about the program that you change, what would it be?)

What did you learn from the program as a mentor?

Did you see your mentoring style change over time? Have you learned something from the facilities that they plan on implementing in their own facilities?

**Closing remarks**

Do you have anything further to add that hasn’t been covered in the interview?

**Interview Protocol – Leaders**

**Background**

1. How many years have you worked at this facility? How many years have you worked in this position?

**Safe Surgery 2020 Initiative Mentoring Intervention**

1. Can you describe the mentorship intervention? (Prompt for SS2020 purpose process, timeline, structure)

**Areas of mentoring support**

1. When the mentorship program began, what was your vision for the program? Has this vision been met?
2. Can you describe the areas of mentoring that the mentors provide to you and your hospital’s surgical team?
   1. In which areas would you say mentors are providing the most support? The least support?
   2. In which areas would you say are the most valuable to your facility? Which areas would you say are the least valuable?

Which type of providers in your facility benefited the most from mentoring? Were people in some roles better suited to mentoring than others? If so, who?

**Perceptions of program, mentors, mentor relationships, and experiences**

1. Can you describe some characteristics that made the mentors in your facility effective?
2. How would you describe the relationships between the mentors and surgical staff?
   1. How comfortable did the mentors seem in their role?
   2. Were mentors confident in their skills?
   3. Were surgical staff members willing to seek help and admit mistakes to mentors?
3. In which ways were you involved in the mentoring process? How do you support it?
4. Can you describe the general attitude around adopting the mentoring program in your facility? (Prompt for acceptance or resistance by providers in specific roles/all providers, culture change, culture of mentoring)

**In person mentoring and Project Echo**

1. Are you familiar with Project ECHO?
2. How does Project ECHO compare to the in-person mentorship?
3. Can you please tell me about the WhatsApp Groups between facilities your staff have been using for peer to peer mentoring? Are you involved in those groups?
   1. What types of things do they discuss on the WhatsApp Group?
   2. What do you find useful about it?
4. What are the opportunities where the different types of mentoring (in person, Project ECHO, peer-to-peer mentoring (WhatsApp Group) can complement one another?

Probes:

1. Which mentoring activities are most effective through in-person mentorship?
2. Which types of mentoring activities are most effective through Project Echo? Peer-to-peer mentoring?
3. Do you think all are needed?
4. Do you think the three types of mentorship should be implemented at the same time or one at a time? If it is the latter, in what order should they be implemented?

**Difference made by mentoring**

1. In what ways do you think the mentoring program is contributing towards strengthening surgical and anesthesia services in your facility? (e.g. Facility benefits, provider skill benefits) *(if already answered by participant, omit from questionnaire)*
2. Has mentorship impacted the team dynamic of your facility? If so, how? (Probe for freedom to ask questions, open communication etc.)
3. How did you assess the impact on mentorship among providers in your facility? Did you speak with the staff members intermittently? Was their feedback taken into consideration in any way? If not, do you have a plan for that in future?
4. What changes have been made at a hospital level (including system processes)
5. Have you seen changes in the surgical team or processes related to surgery?

**Strengths, challenges, areas for improvement, and lessons learned**

1. Has the mentorship program met your expectations? Why or why not?
2. What are the areas in which the program could improve?

a. If you could change one thing about the mentorship program, what would it be?

b. What was the one thing that worked best for you in the program?

How long do you think mentorship should continue for your facility?

**Closing remarks**

1. Do you have any further questions, concerns, or comments for our team regarding the mentorship program?

**Focus Discussion Group Questions**

Opening questions

1. In your own words, can you please describe the Project Echo program?
2. Can you describe the areas of mentoring support received through Project Echo?

Experience with Project Echo

1. Of the areas of mentoring discussed, which are the most valuable and why? (didactic presentation, case presentation, discussion, presenting to colleagues)?
2. Which areas are the least valuable and why?
3. Consider all types of mentoring you have experienced during SS2020, what elements of mentorship can be accomplished through Project ECHO? (Probe: Learning a new skill, presenting progress on action plans, discussing complicated cases or procedures, asking questions to mentors, evaluating data for quality improvement)
4. Are some activities less effective when done through the telementoring platform?
5. What elements of mentorship need to be accomplished in person? (Probe: Which activities do you prefer to do face-to-face?)

Barriers, Facilitators

1. What barriers did you experience in attending Project ECHO sessions? (probe for barriers: internet connection, time of session, transportation to session, workload)
   1. How was the internet connection (or electricity?) during the ECHO sessions? Did this affect your ability to understand what is going on? Your ability to connect with mentors or peers?
2. What strategies can be used to facilitate successful implementation of Project Echo?

Impact

1. In what ways do you think Project Echo is contributing to strengthening surgical and anaesthesia services in your facility?
2. Do you or anyone on your team do anything differently as a result of Project Echo? Can you provide examples?

Sustainability

1. Would you continue using Project ECHO after the SS2020 project is complete?
