# Supplementary material for: A multimodal mentorship intervention to improve surgical quality in Tanzania’s Lake Zone: a convergent, mixed methods assessment
Source: Hum Resour Health. 2021 Sep 23;19:115. doi: 10.1186/s12960-021-00652-6 (PMC8458007; doi:10.1186/s12960-021-00652-6)

**Additional File 3 – Overall experience, overall satisfactio and support for continuation of the mentorship program**


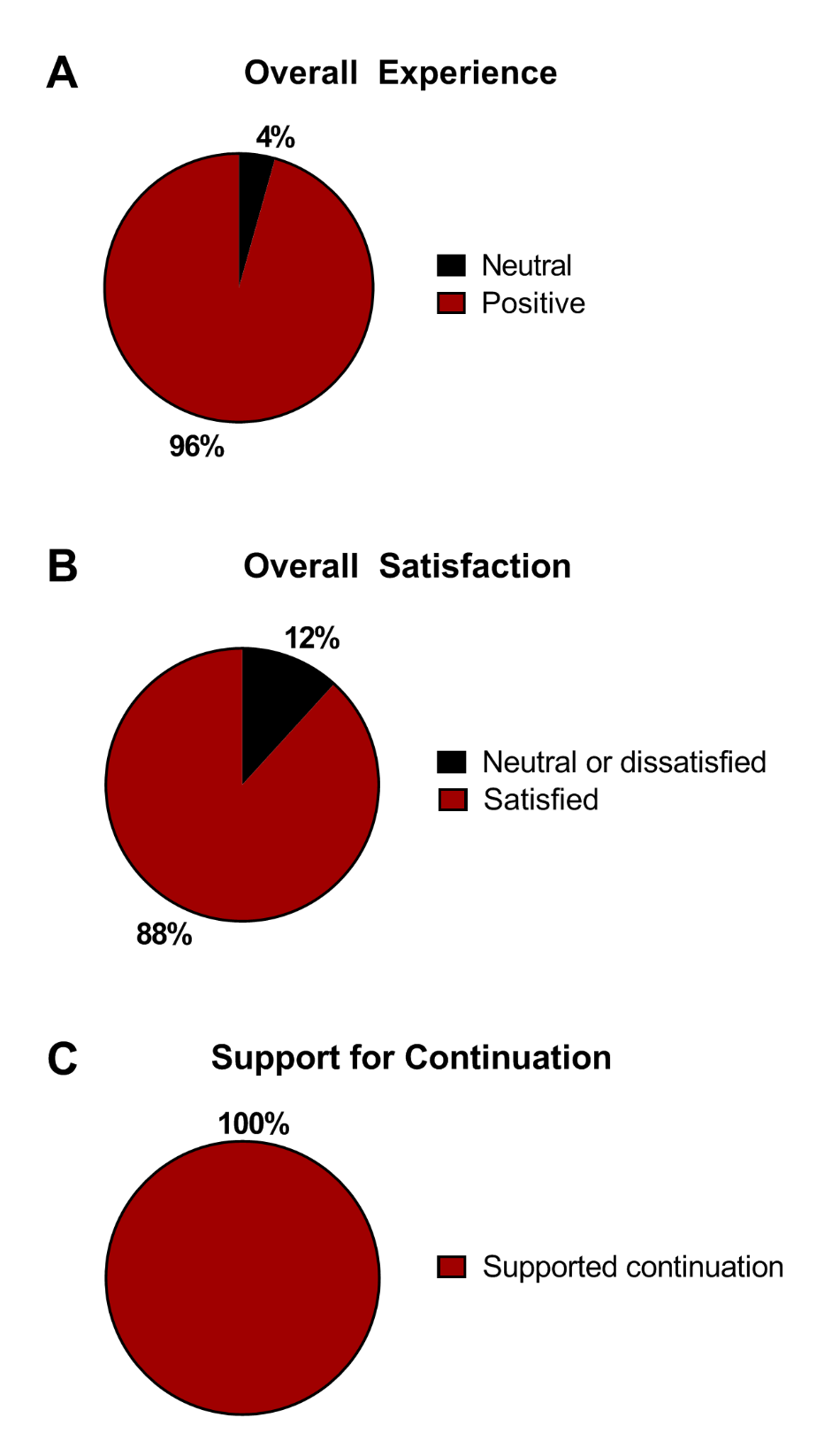

Supplement: Supplementary file 3 — Additional file 3: Overall experience, overall satisfaction and support for continuation of the mentorship program. [file 12960_2021_652_MOESM3_ESM.docx]
