# Supplementary figures and images for: A multimodal mentorship intervention to improve surgical quality in Tanzania’s Lake Zone: a convergent, mixed methods assessment
Source: Hum Resour Health. 2021 Sep 23;19:115. doi: 10.1186/s12960-021-00652-6 (PMC8458007; doi:10.1186/s12960-021-00652-6)

**Additional File 4- Changes made resulting from the mentorship program**


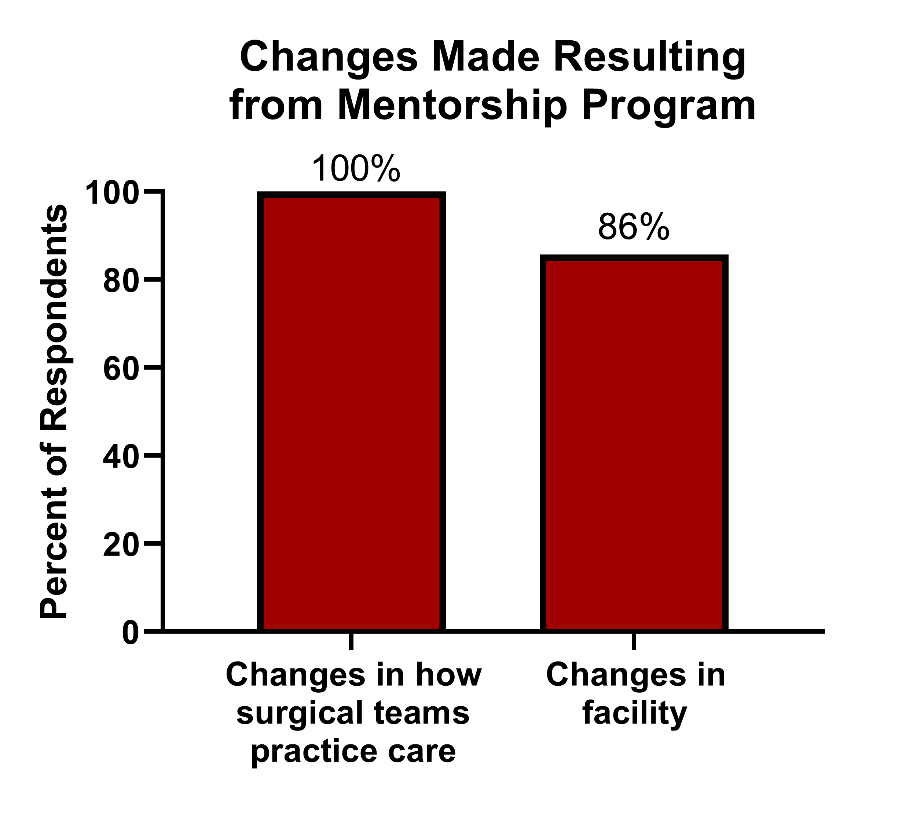

Supplement: Supplementary file 4 — Additional file 4: Changes made resulting from the mentorship program. [file 12960_2021_652_MOESM4_ESM.docx]

**Additional File 5: Areas of Mentorship desired and received by mentees**


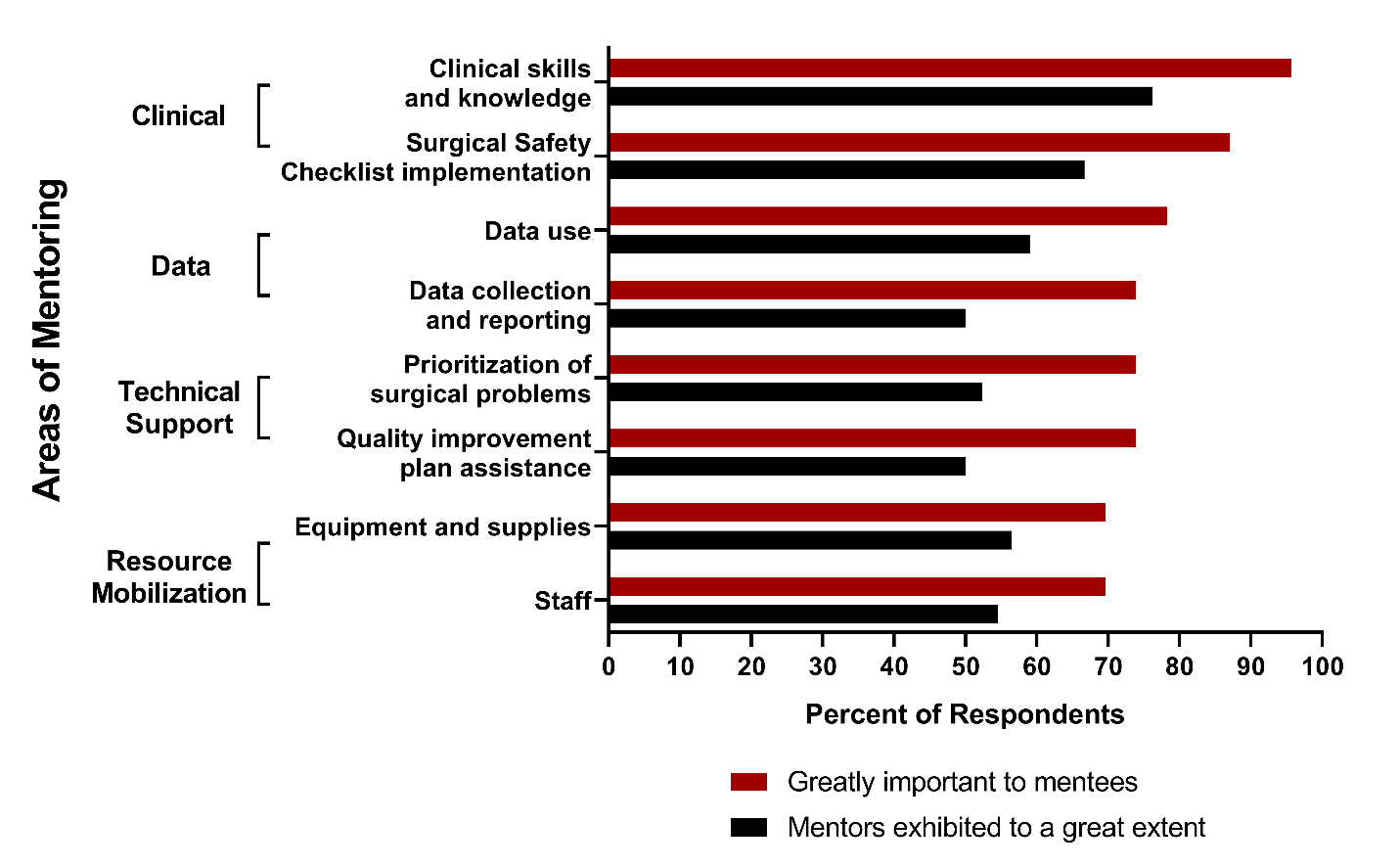

Supplement: Supplementary file 5 — Additional file 5: Areas of Mentorship desired and received by mentees. [file 12960_2021_652_MOESM5_ESM.docx]

**Additional File 6: Mentor characteristics desired and exhibited by mentors**


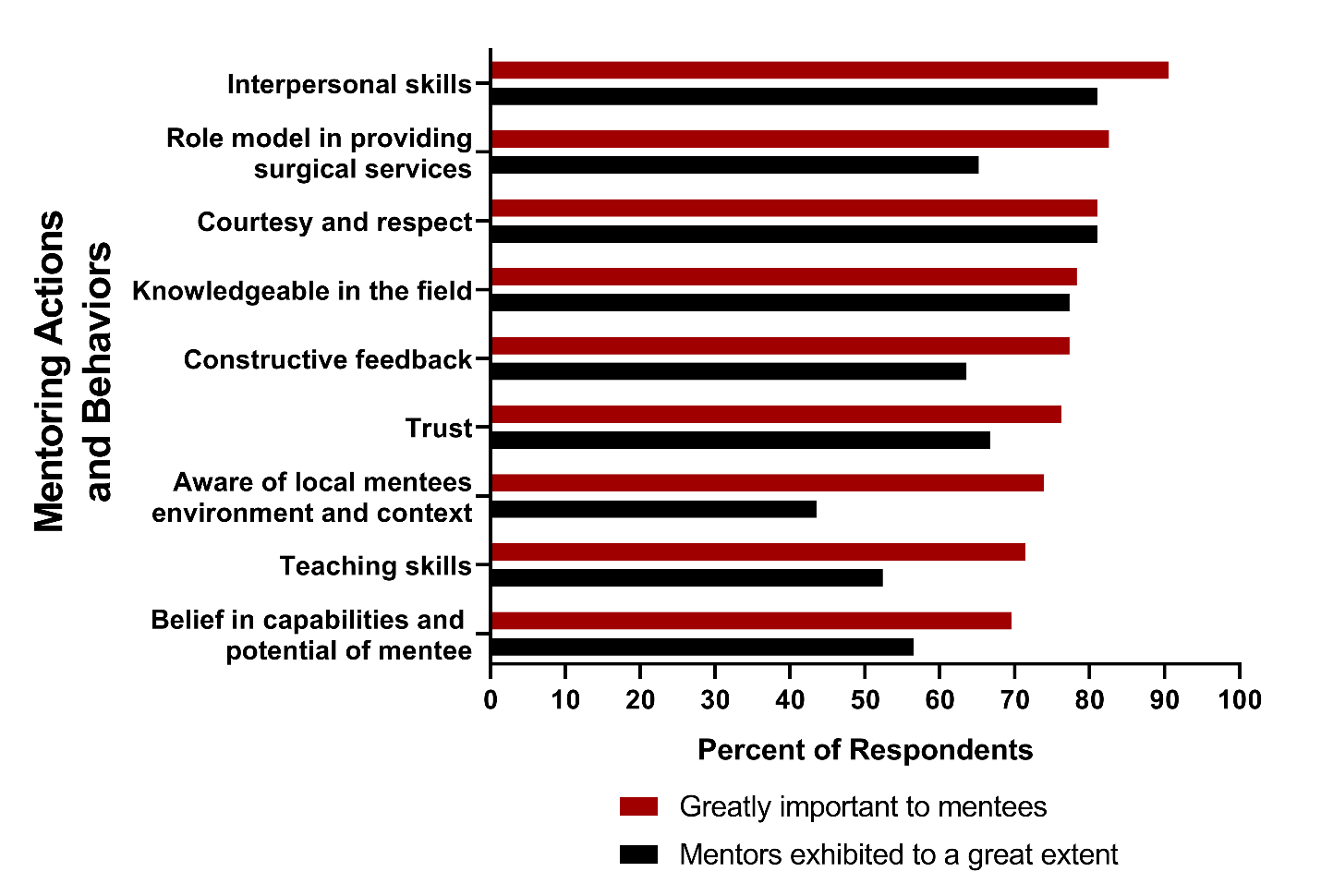

Supplement: Supplementary file 6 — Additional file 6:Mentor characteristics desired by mentees and exhibited by mentors. [file 12960_2021_652_MOESM6_ESM.docx]
